# Emergent Management of Clostridium difficle Infection in a Patient With Chronic Inflammatory Bowel Disease

**DOI:** 10.7759/cureus.14751

**Published:** 2021-04-29

**Authors:** Lauren H Pomerantz, Scott Hewitt

**Affiliations:** 1 Medicine, University of Central Florida College of Medicine, Orlando, USA; 2 General Surgery, MacDill Air Force Base (AFB), Tampa, USA; 3 General Surgery, C. W. Bill Young Veterans Affairs (VA) Medical Center, Bay Pines, USA

**Keywords:** total colectomy, clostridium difficle infection, ulcerative colitis, rectal perforation, general surgery, emergent general surgery

## Abstract

Clostridium difficle (C. diff) is a well known cause of infectious diarrhea across hospitals in the developed world. An anaerobic, gram positive rod bacteria, C. diff is part of the normal flora of the human colon; however, alterations to the microbiome can promote proliferation leading to pathogenic behavior. Typical symptoms include watery diarrhea in excess of three or more times a day, for at least two days, and abdominal cramping. While most infections do not lead to long term complications, the two complications that are most deleterious to health are toxic megacolon and bowel perforation. Patients with an inflammatory bowel disease are at a higher risk of complications, and thus need to be managed appropriately. This case presents a 39-year-old male, with pertinent medical history of poorly controlled ulcerative colitis, who presented to general surgery with imaging suggesting rectal perforation secondary to a C. diff infection. Due to the free air visualized in the rectum, the patient was urgently transported to the operating room to undergo a total colectomy and end ileostomy surgery. This case discusses the well-known complication of bowel perforation, in order to raise awareness about the management and guidelines. This case is important and significant as it details the appropriate guidelines and structure to follow amongst this unique, and vulnerable to complications, population in order to manage a potentially devastating manifestation of C. diff.

## Introduction

There are a few key risk factors that increase one's susceptibility to Clostridium difficle (C. diff), including but not limited to: being a member of an at-risk population, being immunosuppressed, being of advanced age, being malnourished, and having received a recent dose of antibiotics. Regarding antibiotics, one study found that 96% of symptomatic patients with C. diff colitis had received antibiotics within 14 days of infection [1]. Any antibiotic may be the culprit; however, cephalosporins, clindamycin, penicillin, and fluoroquinolones are the most common [1]. Ultimately, however, antibiotics are not required to acquire C. diff.

According to the 2019 Center for Disease Control Antibiotic Resistance Threats report, C. diff is a major health threat acquiring urgent status [2]. It noted that in 2017, there were an estimated 223,900 cases diagnosed in hospitalized patients and 12,800 deaths in the United States related to C. diff infections [2]. Inflammatory bowel disease (IBD) patients pose a higher risk of acquiring a C. diff infection due to the risk of antibiotics coupled with colonic dysbiosis and loss of resistance to bacterial colonization with underlying inflammatory colitis that allows for C. diff bacteria to penetrate [3]. While this antibiotic exposure is the most commonly documented cause of C. diff, it is important to note that C. diff may present spontaneously in people with a known history of IBD. When a patient has known IBD and worsening diarrhea, an important differential is C. diff even if there has not been recent antibiotic usage.

C. diff infections need to be taken seriously with all patients, but when patients have a history of IBD, they pose unique concerns. Between 2004 and 2005, it was found that among IBD patients infected with C. diff, more than half required hospitalization, and 20% required colectomy [4]. Patients with a history of IBD that contract C. diff, carry four times higher mortality rate and three days longer hospital stay than those patients who acquire C. diff without underlying IBD [5]. This stated increase in hospital stay and increase in mortality correlates with the increased risk of complications in IBD patients.

## Case presentation

A 39-year-old male with a five-year history of moderate ulcerative colitis (UC), as per Montreal classification, was admitted to the emergency department with worsening abdominal and rectal pain secondary to a C. diff infection of one week. Patient had a multi-week history of diarrhea that was waking him up at night leading up to the C. diff diagnosis. One week prior to ED admission, his GI had ordered precautionary leukocytes and C. diff due to history of UC, and both returned positive. Per GI note, "Vancomycin prescribed as this patient has IBD and patients with IBD can develop spontaneous C. diff infections and need to be treated regardless of toxin presence." He had no prior history of antibiotic use over the past three months or triggering factor for C. diff infection. 

Over the course of the week, his symptoms progressed, and he claimed to have nausea, vomiting, and hematochezia.

The patient presented with a past medical history of depression, post-traumatic stress disorder, and obstructive sleep apnea. His allergies included trazodone and amoxicillin/clavulanate. His family history was unrevealing. His surgical history included cholecystectomy and thyroglossal duct cyst removal in 2019.

This patient was a nonsmoker. He denied alcohol or drug use. He tried to maintain a healthy diet of lean proteins, fruits, and vegetables.

Initial vitals on presentation included: temperature: 98.6 F, pulse: 90 beats per minute, respiratory rate: 15 breaths per minute, pulse oximetry: 95%, blood pressure: 119/87 mmHg, weight: 217.16 lbs, and body mass index: 32.14.

**Figure 1:**
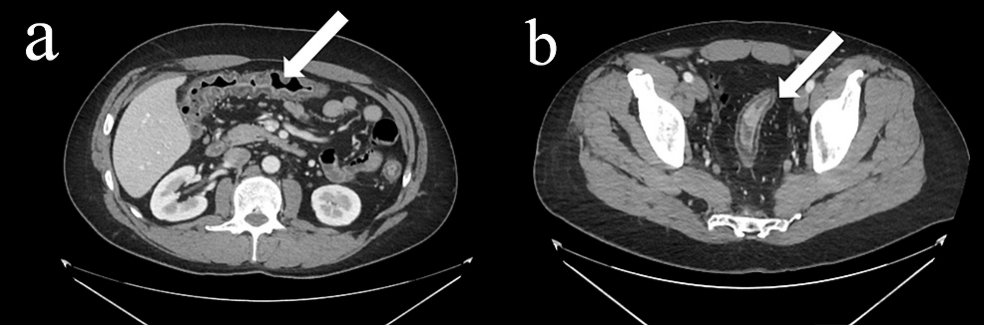
Axial Computerized Tomography (CT) images of Abdomen/Pelvis With Contrast (a) Axial computerized tomography scan images of abdomen/pelvis with contrast indicating shortening and loss of haustra, as well as submucosal fat infiltration consistent with colonic inflammation and ulcerative colitis. (b) Axial computerized tomography scan images of abdomen/pelvis with contrast indicating shortening and loss of haustra further down the abdomen indicating complete consumption of colon with inflammation.

**Figure 2:**
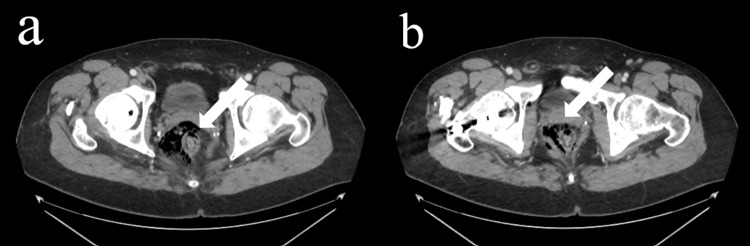
Axial Computerized Tomography (CT) images of Abdomen/Pelvis With Contrast (a) Axial computerized tomography scan images of abdomen/pelvis with contrast indicating new extraluminal gas in the perineum surrounding anus and distal rectum with findings indicating anteromedial perforation. (b) Axial computerized tomography scan images of abdomen/pelvis with contrast indicating free air.

General surgery was consulted upon CT scan with IV contrast revealing new extraluminal gas in the perineum surrounding the anus and distal rectum (Figures 1a, 1b, 2a, 2b). He was examined and subsequently diagnosed with rectal perforation.

Preoperatively, the patient’s physical exam revealed an uncomfortable looking young man in mild distress due to abdominal pain. An abdominal exam revealed no distention, masses, rebound, or guarding. The absence of McBurney’s sign, Murphy’s sign, and peritoneal inflammation was noted. There was no active rectal bleeding at the time of physical examination. Laboratory data revealed an elevated c-reactive protein of 66.4. Complete blood count and complete metabolic panel were otherwise within normal limits. Despite infection and imaging reflecting perforation, white blood count was found to be 5.9 k/cmm.

After review of the available data, assessment of clinical condition, and deliberation with the emergency department physicians and the patient’s primary gastroenterology physician, the surgery team decided the patient would require an urgent exploratory laparotomy with total abdominal colectomy and end ileostomy due to the diagnosis of rectal perforation secondary to C. diff colitis on chronic UC.

The patient was immediately brought to the operating room. The primary incision was made from the subxiphoid to the pubic symphysis within two hours of initial presentation to the hospital. The colon was visualized and resected; thus, a total colectomy was undergone. The pre-rectal space was opened to identify signs of purulence or injury, which found crepitus and severe UC causing circumferential ulcerations and a deep ulcer in the midline anteriorly. These ulcers traversed all layers of the rectal wall. A drain was placed to drain potential abscess or infection from the pre-rectal space.

The patient left the OR in stable condition after enduring no complications intraoperatively. He remained in the inpatient surgical unit for five days, during which he continued to advance his diet and ambulate successfully. He was discharged after an education session on ostomy bag maintenance and told to follow up with the general surgery team and gastroenterology team in one week. At his one-week follow-up post-op visit, the patient was doing well, ostomy output was draining appropriately, and he denied fever, nausea, or vomiting. No secondary follow-up surgery was required.

## Discussion

The management of C. diff in IBD patients follows standard guidelines, but many providers, both specialists and generalists, might not be acutely aware of protocol. IBD colitis flare-ups often present with the same clinical presentation as C. diff infections, both producing copious amounts of diarrhea and severe abdominal pain. Therefore, the American College of Gastroenterology outlines that all IBD patients undergoing a suspected flare must also be tested for C. diff colitis. Glutamate dehydrogenase enzyme sampling followed by stool cultures in conjunction with cell cytotoxicity assays testing for toxins A and B are the accepted measures of testing [1].

Once diagnosed with a C. diff infection, initial safety precautions are essential as follows: (1) halting the initial, causative antibiotic if there is one and (2) implementing decontamination methods of handwashing and isolation. To target the pathogen itself, oral metronidazole, vancomycin, or fidaxomicin are first-line treatments, as approved by the federal drug administration in 2011 [6]. Fecal microbiome transplants have shown success in the management of C. diff infections in non-IBD patients, reporting near 94% eradication of the disease after two infusions of donor feces [6]. IBD patients have not had confirmation of success, and therefore, it is not currently approved to try that method in this patient population.

Medication management is often able to clear the underlying infection; however, 1% of patients with C. diff without comorbidities and 30% of those with severe disease comorbidities ultimately require emergency surgery [1]. Surgery is considered standard of care for patients with severe colitis who fail to improve with medical therapy, those that develop peritonitis, those that develop a colonic perforation, or those that develop toxic megacolon [1]. Eighty-nine percent of patients that require surgery undergo a total colectomy with end ileostomy, as seen in this case, with the remaining following various other protocols of segmental colectomy. It should be noted that patients undergoing partial resection needed further resection through a second operation at a later date 16% of the time [1]. The procedure of diverting loop ileostomy with colonic lavage is an option; however, there is weak research-based support on the technique's success.

In this case, the standard guidelines were followed, beginning with immediate initiation of oral vancomycin and self-isolation, concluding with a total colectomy and end ileostomy after complication.

## Conclusions

This case highlights a unique case of spontaneous C. diff infection in patients with a history of IBD. When managing an infected patient with IBD, precautions of early antibiotic therapy should be taken to attempt to minimize the risk of harmful complications of C. diff; however, even if precautions are taken, it is still possible to develop perforation. If an IBD patient is to fall ill with the infection, standard management follows the protocol of decontamination, isolation, and antibiotic use. If all medical management fails, or there is a further complication, then total surgical colectomy should be the last resort.
